# Recurrent right ventricular cardiac myxoma in a patient with Carney complex: a case report

**DOI:** 10.1186/1752-1947-8-134

**Published:** 2014-05-02

**Authors:** Muhammad Rizwan Sardar, Ankush Lahoti, Amanulla Khaji, Wajeeha Saeed, Khawar Maqsood, Harry G Zegel, Jeanine E Romanelli, Frank C McGeehin

**Affiliations:** 1 Department of Medicine, Lankenau Medical Centre, Wynnewood, Pennsylvania, USA; 2 Department of Cardiology, Lankenau Medical Centre, Wynnewood, Pennsylvania, USA; 3 Department of Radiology, Lankenau Medical Centre, Wynnewood, Pennsylvania, USA; 4 Department of Cardiology, Cleveland Clinic Foundation, Weston, Florida, USA; 5 Department of Medicine, Albert Einstein College of Medicine, Bronx, New York, USA; 6 Department of Medicine, Baystate Medical Centre, Springfield, Massachusetts, USA; 7 Hospitalist office, second floor, Heart pavilion, Lankenau Medical Center, 100 East Lancaster Ave, 19096 Wynnewood, Pennsylvania, USA

**Keywords:** Carney complex, Pituitary adenoma, Recurrent cardiac myxoma, Right ventricular myxoma

## Abstract

**Introduction:**

Carney complex is a multiple neoplasia syndrome involving cardiac, endocrine, neural and cutaneous tumors with a variety of pigmented skin lesions. It has an autosomal dominant mode of inheritance. Approximately 7% of cardiac myxomas are related to the Carney complex. Myxomas that occur as part of the Carney complex affect both sexes with equal frequency. Cardiac myxomas with Carney complex are reported mostly in the left side of the heart and are less common on the right side. As per our review, this is the first reported case of Carney complex with right ventricle cardiac myxoma.

**Case presentation:**

We present a rare case of recurrent cardiac myxoma in a patient later diagnosed to have Carney complex. A 46-year-old Caucasian man with a history of thyroid hyperplasia came to out-patient cardiology department with new onset atrial fibrillation. A transthoracic echocardiogram revealed a right ventricular mass attached to his interventricular septum, which was later seen on a transesophageal echocardiogram and cardiac magnetic resonance imaging. He underwent resection of the ventricular mass which on pathology revealed myxoma. He later developed skin lesions, pituitary adenoma and Sertoli cell tumor suggesting Carney complex. Two years later he developed a new mass within his right atrium which was later resected.

**Conclusions:**

Carney complex is a rare autosomal dominant disease with variable penetrance. Since it involves multiple organs, patients diagnosed with Carney complex should undergo serial endocrine workup, neural assessments, echocardiograms and testicular ultrasounds. Of the total number of cases of Carney complex, 65% are linked to *PRKAR1A* gene mutation. It is important for clinicians to be cognizant of a link between cardiac myxoma and Carney complex. The use of multi-imaging modalities allows better delineation of the mass before planned resection. Carney complex-related cardiac myxoma comprises 7% of all cardiac myxomas. Right ventricular cardiac myxomas are rare. This case report is the first to describe right ventricular myxoma with Carney complex.

## Introduction

Carney complex (CNC) is an autosomal dominant multiple neoplasia syndrome involving skin and cardiac myxomas, pigmented skin lesions and endocrine tumors. About 700 patients worldwide have been reported by the National Institutes of Health, Mayo Clinic (USA) and the Institut Cochin (France) by January 2008 [1]. Cardiac myxomas are the second most common manifestations of CNC after skin lentigines [2]. Among all cardiac myxomas, left atrial cardiac myxomas are the most common (75%) [3,4], followed by right atrial (18%), with left and right ventricular myxomas being the least common (2.5 to 4%) [5-7]. Nearly 7% of all cardiac myxomas are associated with CNC. Various locations of cardiac myxomas associated with CNC have been reported so far but none originating in the right ventricle. We are reporting a case of recurrent cardiac myxoma of the right side of the heart which recurred 2 years after the initial presentation with simultaneous pituitary microadenoma and skin lentigines consistent with the diagnosis of CNC.

## Case presentation

**Figure 1:**
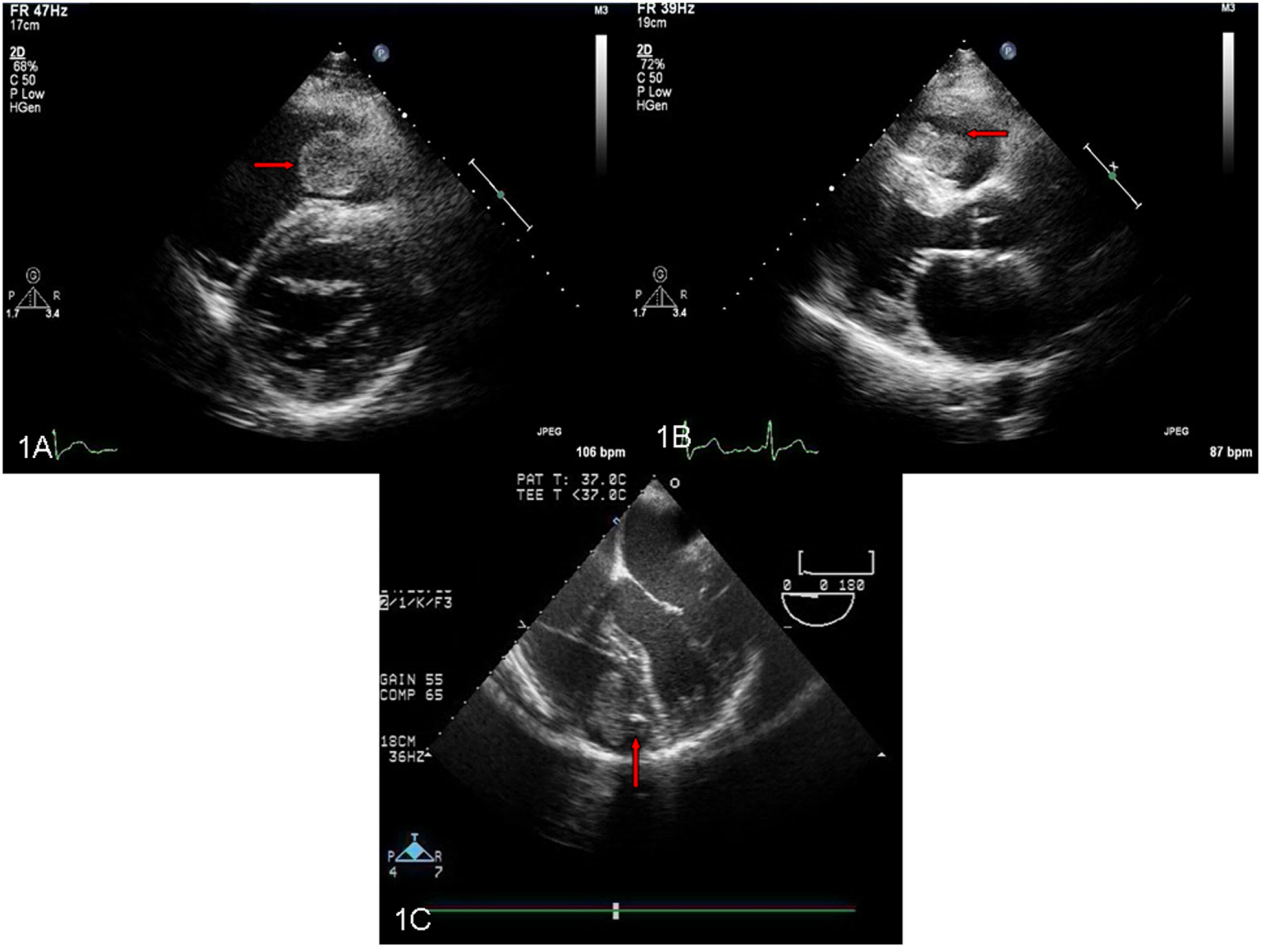
**Transthoracic and transesophageal echocardiograms. (A, B)** Parasternal long and short axis views showing right ventricular mass (marked with red arrow). **C**: Transesophageal echocardiogram: Five chamber view, showing right ventricular mass attached with a stalk to interventricular septum (red arrow).

**Figure 2:**
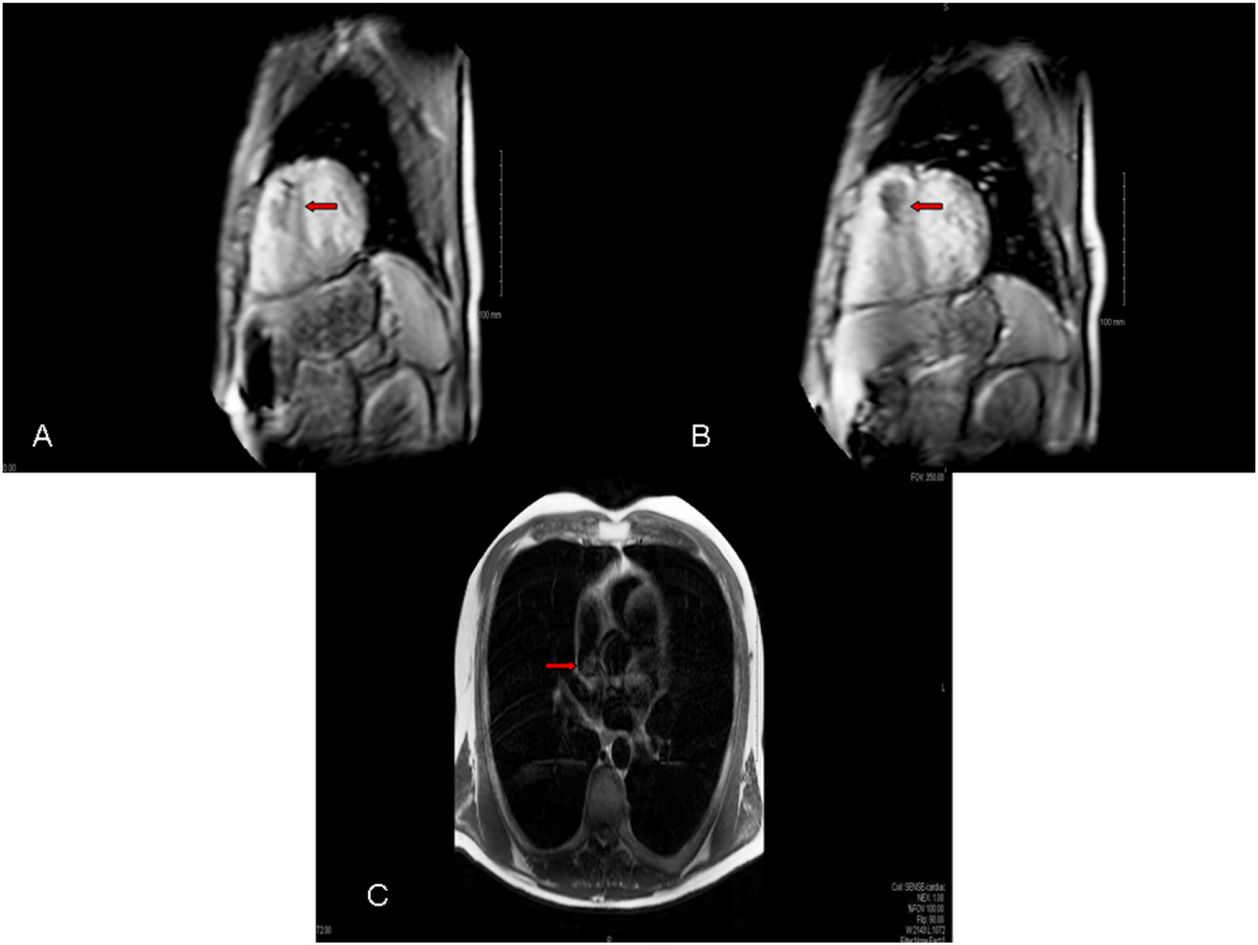
**Magnetic resonance imaging. (A, B)** Magnetic resonance imaging perfusion imaging showing right ventricular mass extending from the base of the right ventricle to the apex (red arrow). **C**: Magnetic resonance imaging pre-scan (pre-perfusion) images showing right ventricular mass (red arrow).

A 46-year-old Caucasian man recently diagnosed with benign thyroid hyperplasia presented to his primary care physician with palpitations, light-headedness, and dizziness and was found to be in atrial fibrillation with rapid heart rate of 140 beats per minute. He denied chest pain, shortness of breath, fevers, night sweats, weight loss, and fatigue. His heart rate was controlled and a transthoracic echocardiogram (TTE) was obtained which showed a right ventricular mass attached to his interventricular septum that was confirmed on a subsequent transesophageal echocardiogram (Figure 1A, 1B, 1C) [Additional file 1: Movies 1 and 2]. Cardiac magnetic resonance imaging (MRI) showed a 2.5cm soft tissue density in his right ventricle attached to the mid-septum (Figure 2A, 2B, 2C).

**Figure 3:**
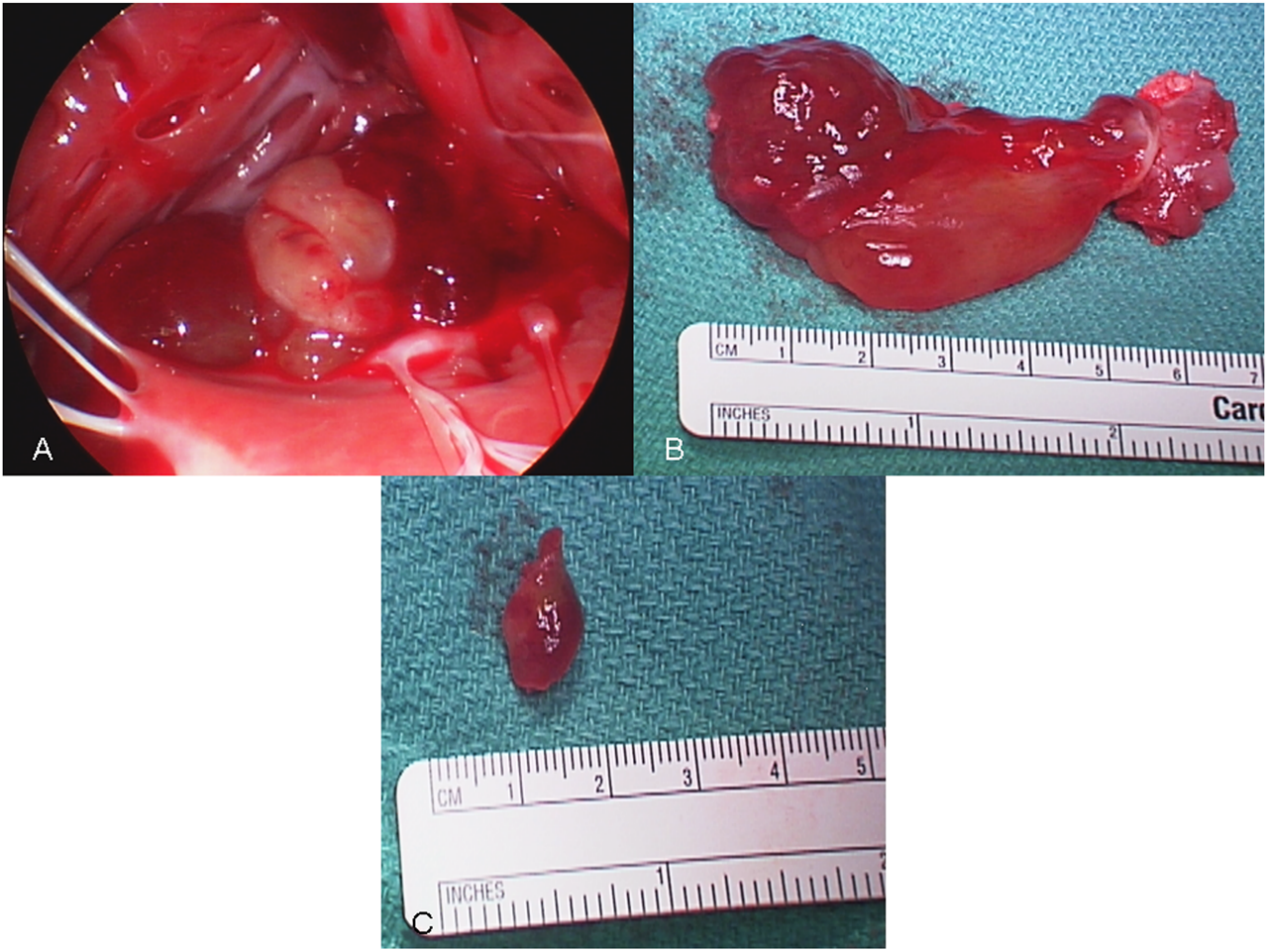
**Intra- operative pictures. A:** A right ventricular myxoma seen through the tricuspid valve intraoperatively. **B**: Right ventricular mass attached to the base of the right ventricle. **C**: Small resected mass at the apex of right atrium.

**Figure 4:**
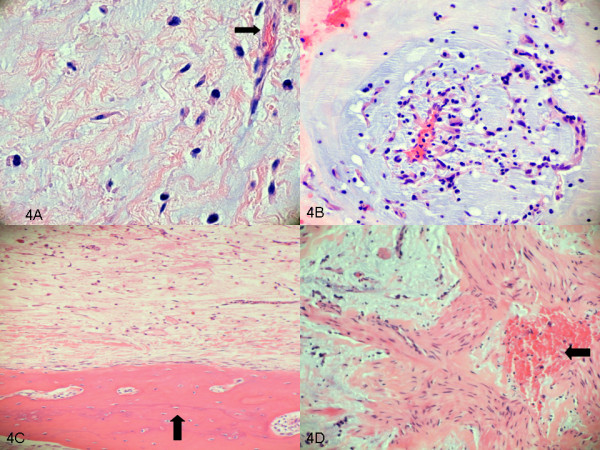
**Histopathology. A**: Hematoxylin and eosin stain showing scattered single myxoma cells, stellate and plump, in a background of amorphous; basophilic material admixed with fibrillary pink stroma. **B**: Hematoxylin and eosin stain showing myxoma cells forming rings, cords and nests. **C**: Hematoxylin and eosin stain showing heterologous bone within tumor. **D**: Hematoxylin and eosin stain showing heterologous smooth muscle elements within the tumor.

A surface echocardiogram was performed intraoperatively [Additional file 2: Movie 3] and surgical resection of the right ventricular mass was performed followed by the Maze procedure. The resected mass was bilobed measuring at least 7cm×1.5cm (Figure 3A, 3B). Pathology confirmed a myxoma (Figure 4A, 4B, 4C, 4D). Brownish macules were noted on the patient’s lips, conjunctivae and all over his body. Considering his examination findings, cardiac myxoma and history of thyroid neoplasm, concern for a genetic syndrome was raised. He denied any known family history of similar examination findings or known hereditary diseases. At that time he did not pursue genetic screening and was followed regularly with yearly TTE.

Screening with TTE 2 years later revealed a new mass in his right atrium measuring 1.8cm×1.4cm on resection and it was a myxoma (Figure 3C). He underwent rigorous workup for CNC which revealed elevated insulin-like growth factor 1 (IGF-1) levels, and positive oral glucose tolerance test with pituitary microadenoma on MRI consistent with acromegaly. He was also diagnosed with bilateral multiple large-cell calcifying Sertoli cell tumor (LCCSCT) of the testes. He was treated for acromegaly and was also recommended to follow up with repeat pituitary MRI in 6 months and an annual testicular ultrasonography. His children were sent for genetic studies.

## Discussion

The CNC was first described in 1985 by J. Aidan Carney, as the combination of myxomas, spotty pigmentation and endocrine overactivity [8]. CNC is defined as an autosomal dominant, multiple neoplasia syndrome involving skin and cardiac myxomas, pigmented skin lesions and endocrine tumors.

The most recent diagnostic criteria include clinical findings such as spotty skin pigmentation, cutaneous and cardiac myxomas, breast myxomatosis, acromegaly, primary pigmented nodular adrenocortical disease, blue nevus, epithelioid blue nevus, osteochondromyxoma, thyroid carcinoma, LCCSCT in males, ovarian cyst in females and mutation of the *PRKAR1A* gene. At least two of these manifestations must be present to confirm the diagnosis of CNC. With *PRKAR1A* mutation and/or a first-degree relative affected by CNC, a single manifestation is sufficient for the diagnosis [8-11].

Cardiac myxomas are rare benign tumors accounting for 45% of primary cardiac tumors in adults [12] and among cardiac myxomas, left atrial cardiac myxomas are the most common (75%) followed by right atrial (18%), followed by left and right ventricular myxomas being the least common (2.5 to 4%). Up to 7% of all the cardiac myxomas are associated with CNC and are notorious for recurrence [13] when compared to sporadic cardiac myxomas. CNC cardiac myxomas are known to have a left heart origin and are unlikely to be right sided as in our patient. Patients with CNC who have cardiac tumors may be totally asymptomatic but have an increased risk of sudden death, which makes it clinically important.

CNC is associated with at least two genetic loci including the *PRKAR1A* gene located on chromosome 17 and the *CNC2* locus mapped to chromosome 2 [14] and a possible third loci. Clinical suspicion of CNC should be followed by thorough evaluation. This includes biochemical and imaging modalities for endocrine, cardiac and skin tumors. A preoperative coronary angiograph is advised if coronary artery disease is suspected or if the patient is above the age of 40 years [15]. Surgical excision of the tumors is the primary management of most tumors [16].

Due to its high association with morbidity and mortality, surveillance is key. For pre-pubertal, post-pubertal children and adults with excised myxoma, an echocardiogram is performed every 6 months or yearly. For children with LCCSCT, close monitoring of growth rate, pubertal status and testicular ultrasonography are essential. Every patient diagnosed with CNC should have a yearly testicular ultrasound, and serum IGF-1 levels and urinary free cortisol levels should be monitored. Thyroid ultrasound and ovarian ultrasound should be repeated as needed [17]. Approximately 70% of individuals diagnosed with CNC have an affected parent; approximately 30% have a *de novo* mutation [1]; first degree relatives should be screened when *PRKAR1A* mutations are identified.

## Conclusions

Our patient initially had multiple myxomas in his right ventricle, the least common location of cardiac myxomas, which aroused the suspicion of CNC and reevaluation confirmed the diagnosis. This is the first case of right ventricular cardiac myxoma in association with CNC.

## Consent

Written informed consent was obtained from the patient for publication of this case report and any accompanying images. A copy of the written consent is available for review by the Editor-in-Chief of this journal.

## Abbreviations

CNC: Carney complex; IGF-1: Insulin-like growth factor 1; LCCSCT: Large-cell calcifying Sertoli cell tumor; MRI: Magnetic resonance imaging; TTE: Transthoracic echocardiogram.

## Competing interests

The authors declare that they have no competing interests.

## Authors’ contributions

AL, WS and KM carried out manuscript writing. AK and WS carried out editing of manuscript and images. JER, FCM and HGZ carried out proofreading of the manuscript. MRS carried out manuscript design, conceptualization, manuscript writing, editing and final proofread. All authors read and approved the final manuscript.

## Supplementary Material

Additional file 1 **Movie 1 and 2.** Transthoracic echocardiogram and transesophageal echocardiogram showing right ventricle mass. Click here for file

Additional file 2 **Movie 3.** Surface echocardiogram in the operating room reveals bilobed right ventricular mass. Click here for file
